# Impact of Cognitive VR vs. Traditional Training on Emotional Self-Efficacy and Cognitive Function in Patients with Multiple Sclerosis: A Retrospective Study Focusing on Gender Differences

**DOI:** 10.3390/brainsci14121227

**Published:** 2024-12-05

**Authors:** Maria Grazia Maggio, Alessandra Benenati, Federica Impellizzeri, Amelia Rizzo, Martina Barbera, Antonino Cannavò, Vera Gregoli, Giovanni Morone, Francesco Chirico, Angelo Quartarone, Rocco Salvatore Calabrò

**Affiliations:** 1 IRCCS Centro Neurolesi Bonino-Pulejo, 98124 Messina, Italy; mariagrazia.maggio@irccsme.it (M.G.M.); angelo.quartarone@irccsme.it (A.Q.); roccos.calabro@irccsme.it (R.S.C.); 2 Department of Psychology “Renzo Canestrari”, University of Bologna, Via Zamboni, 33, 40126 Bologna, Italy; abenenati74@gmail.com; 3 Department of Clinical and Experimental Medicine, University of Messina, Piazza Pugliatti, 1, 98122 Messina, Italy; amrizzo@unime.it (A.R.); martinabarbera17@gmail.com (M.B.); 4 A.O.U. Policlinico “G. Martino”, Via Consolare Valeria, 98124 Messina, Italy; antonino.cannavo.85@gmail.com; 5 Department of Biomedicine and Prevention, Tor Vergata University of Rome, Via Cracovia, 90, 00133 Roma, Italy; veragregoli@gmail.com; 6 Department of Life, Health and Environmental Sciences, University of L’Aquila, 67100 L’Aquila, Italy; giovanni.morone@univaq.it; 7 San Raffaele Institute of Sulmona, 67039 Sulmona, Italy; 8 Post-Graduate School of Occupational Medicine, Università Cattolica del Sacro Cuore, 00168 Rome, Italy; medlavchirico@gmail.com

**Keywords:** multiple sclerosis, neurorehabilitation, virtual reality, emotional self-efficacy, cognitive rehabilitation, gender differences

## Abstract

Background and aim: While conventional MS rehabilitation primarily addresses physical and cognitive symptoms, recent advances in VR technology offer immersive environments that facilitate both emotional and cognitive skill development. The purpose of this study is to evaluate the impact of VR-based training on emotional self-efficacy in multiple sclerosis (MS) patients and examine its association with cognitive function improvement. Additionally, this study aims to explore potential gender differences in these outcomes, hypothesizing that gender may influence the effectiveness of VR-based rehabilitation, which could inform more tailored approaches for emotional and cognitive rehabilitation in MS. Method: The present retrospective study analyzed data from 43 MS patients undergoing cognitive and behavioral rehabilitation at the IRCCS Centro Neurolesi “Bonino Pulejo” in Italy, comparing a VR intervention group (VR-G) and a control group receiving traditional rehabilitation. Emotional self-efficacy, depression, and anxiety were assessed, alongside cognitive function pre- and post-intervention. Results: Findings indicate that the VR-G showed significant improvements in managing negative emotions, reduced depressive and anxiety symptoms, and enhanced cognitive performance, particularly in verbal learning and working memory. Gender-based analysis revealed a trend suggesting that females in the VR-G may exhibit greater improvements in positive emotional self-efficacy, although this difference did not reach statistical significance. Spearman’s correlation highlighted associations between emotional self-efficacy and cognitive gains, supporting the potential of VR to foster both emotional and cognitive resilience. Conclusions: These findings suggest that VR training may provide a tailored approach for MS rehabilitation, enhancing therapeutic outcomes by integrating emotional and cognitive training in an immersive setting. Further research should investigate long-term effects and neurophysiological correlates of VR training to optimize MS rehabilitation.

## 1. Introduction

Multiple sclerosis (MS) is a chronic autoimmune disease characterized by progressive neurodegeneration, resulting in physical, cognitive, and emotional impairments [1]. The variability in symptoms reflects the disease’s profound impact on the central nervous system as well as patients’ quality of life [2,3]. Epidemiological data indicate that MS affects approximately 2.8 million people worldwide, with a higher prevalence among women, who are two to three times more likely to have the disease than men [4]. This gender disparity highlights the need for research focusing on sex-based differences in disease progression, symptom management, and therapeutic response.

Gender-specific analyses have identified critical differences in MS symptomatology, particularly in the cognitive and emotional domains. Women with MS often report higher levels of depression, anxiety, and emotional dysregulation compared to men [5]. These findings suggest that emotional self-efficacy (i.e., the ability to manage one’s emotional responses effectively) is a key determinant of quality of life, helping in disease management [6]. As the disease progresses, many patients experience cognitive decline, particularly in memory, attention, and executive functions, which may exacerbate emotional challenges [7]. The bidirectional relationship between cognitive and emotional function underscores the need for holistic interventions addressing both domains simultaneously.

Standard MS treatments involve pharmacological and rehabilitative approaches aimed at alleviating physical and cognitive symptoms while supporting emotional well-being [8]. While conventional therapies provide essential benefits, recent advances in virtual reality (VR)-based rehabilitation offer innovative opportunities for cognitive and emotional training. VR is an immersive, controlled environment where patients can engage in targeted cognitive exercises and interact with complex emotional stimuli [9]. In MS rehabilitation, VR training has gained attention for its potential to improve not only physical mobility but also cognitive function and emotional resilience [10].

Focusing on emotional self-management, VR presents distinct advantages. Through VR, patients can safely practice responses to emotionally charged situations, potentially enhancing their self-efficacy in real-life scenarios [11]. Emotional self-efficacy is particularly critical in MS, as individuals with higher confidence in managing their emotions report better psychological outcomes and greater resilience [12]. However, despite the promise of VR in addressing cognitive and emotional challenges, research on gender-specific effects within VR interventions remains limited.

This study aims to evaluate the impact of VR-based training on emotional self-efficacy in MS patients as well as its association with cognitive function improvement. Additionally, it seeks to explore potential gender differences in VR-based rehabilitation outcomes. Findings could support more tailored approaches for emotional and cognitive rehabilitation in MS.

## 2. Materials and Methods

### 2.1. Study Design and Data Source

This retrospective study analyzed data from patients with MS who underwent cognitive and behavioral rehabilitation at the Robotic and Behavioral Neurorehabilitation Unit of IRCCS Centro Neurolesi “Bonino Pulejo”, Messina, Italy, from March 2019 to March 2020. Data were retrieved from medical records, which included assessments conducted at baseline (T0) and after the 8-week rehabilitation program (T1). During this program, participants completed three weekly sessions, each lasting 60 min, as per our standard clinical protocol.

The study adhered to the principles of the 1964 Helsinki Declaration and received approval from the local Ethics Committee (IRCCS-ME-CE 08/21). All participants provided written informed consent for their data to be used for research purposes.

The retrospective design of the study minimized potential scoring bias by relying on pre-existing medical records. Cognitive and behavioral parameters were used to identify eligible MS patients who had participated in both conventional and VR-based rehabilitation programs.

### 2.2. Patient Selection

Patients with MS were included in the study if they met the following inclusion criteria: (i) a diagnosis of MS according to McDonald’s criteria [13]; (ii) stable therapy for at least six months before the start of rehabilitation; (iii) mild to moderate cognitive impairment, defined by a score between 18 and 27 on the Montreal Cognitive Assessment (MoCA) [14]; and (iv) a disability score below 5 on the Expanded Disability Status Scale (EDSS) [15]. Exclusion criteria included patients over 77 or under 18 years of age, those with severe medical or psychiatric conditions likely to interfere with assessments, or those who had experienced an MS relapse within six months preceding the study. A total of 43 patients met these eligibility criteria.

### 2.3. Outcome Measures

The primary outcomes included measures of emotional self-efficacy as well as depressive and anxiety symptoms. Emotional self-efficacy was evaluated using the Scale of Perceived Self-Efficacy in the Management of Negative and Positive Emotions, developed by Caprara and Gerbino. This tool evaluates participants’ confidence in managing both positive (7 items) and negative (8 items) emotions. It demonstrates high internal consistency, with Cronbach’s alpha values typically ranging from 0.80 to 0.90 [16].

Depressive symptoms were measured using the Beck Depression Inventory (BDI), a 21-item self-report inventory widely recognized for quantifying depressive symptom severity. Each item is rated on a scale from 0 to 3, with total scores ranging from 0 to 63, where higher scores indicate more severe depressive symptoms. The BDI consistently demonstrates excellent reliability, with Cronbach’s alpha generally reported to range between 0.86 and 0.92 across populations [17].

Anxiety symptoms were evaluated with the Hamilton Rating Scale for Anxiety (HRS-A), a clinician-administered tool consisting of 14 items that assess both psychological and somatic aspects of anxiety. Each item is rated on a scale from 0 to 4, with higher total scores reflecting greater anxiety severity. This tool has been validated extensively, with Cronbach’s alpha reported to range from 0.77 to 0.92 in various samples [18].

The secondary outcome focused on cognitive function, which was assessed using the Brief Repeatable Battery of Neuropsychological Tests (BRB-N) [19]. This battery evaluates critical cognitive domains, such as verbal and visuospatial memory, attention, and executive functions, providing a comprehensive overview of cognitive performance in neurological conditions. The BRB-N subtests deliver detailed insights into specific cognitive abilities, which are essential for monitoring changes over time in MS patients.

### 2.4. Rehabilitation Protocol

The study included 43 MS patients, divided into two groups. The experimental group (VR-G) consisted of 23 patients who underwent an advanced rehabilitation pathway using the Virtual Reality Rehabilitation System (VRRS). The control group (CG) included 20 patients who received traditional cognitive rehabilitation through conventional methods. Both groups participated in an 8-week rehabilitation program, with three sessions per week, each lasting 60 min. This structure facilitated direct comparisons between the innovative VR-based approach and standard neurorehabilitation methods. Additional details are provided in Table 1.

#### 2.4.1. VR Group

The experimental rehabilitation training incorporated cognitive rehabilitation using VRRS, a medically certified system customized to meet the individual needs and capabilities. The tool facilitated interactive cognitive exercises through 2D virtual scenarios displayed on a semi-immersive screen, consisting of a large LCD monitor positioned at eye level to maximize engagement and focus. Patients interacted with these scenarios using a magnetic tracking sensor, enabling dynamic tasks designed to stimulate cognitive functions such as attention, memory, and problem-solving. For instance, one exercise involved guiding a bear through a forest to collect honey while avoiding obstacles such as branches (requiring quick decision-making, spatial awareness, and sustained attention). This semi-immersive approach provided a controlled, adaptive environment that balanced stimulation and accessibility for all participants [20].

Indeed, the tasks were progressively adapted based on each patient’s performance, ensuring that the difficulty level remained appropriately challenging throughout the intervention.

Rehabilitation sessions were conducted three times a week, with each session lasting approximately 60 min. The consistency and structure of the VRRS sessions provided a stable framework for cognitive rehabilitation while maintaining patient engagement and motivation.

#### 2.4.2. Control Group (CG)

The CG served as the baseline for comparison, reflecting standard rehabilitation care for MS patients without the integration of advanced technologies. Patients in the CG underwent traditional cognitive rehabilitation, using conventional paper-and-pencil tasks. These tasks aimed to improve cognitive functions, including memory, attention, language, and executive functions through established neuropsychological methods. Sessions for the CG were also conducted three times a week, with each session lasting approximately 60 min, matching the frequency and duration of the VR-G rehabilitation. Patients in the CG were selected based on their inability or unwillingness to participate in technology-based interventions due to factors such as contraindications (e.g., musculoskeletal limitations, personal preferences, or a general preference for standard Neurorehabilitation methods. Both groups were comparable at baseline across clinical and physical measures, including the Expanded Disability Status Scale (EDSS), which showed no significant difference between the VR and control groups (*p* = 1.00, Table 2).

### 2.5. Statistical Analysis

All statistical analyses were conducted using IBM SPSS Statistics, version 28, with a significance level set at *p* < 0.05 for all tests. Categorical and ordinal variables were presented as frequencies and percentages, while continuous variables were summarized as medians and interquartile ranges (Q1 and Q3).

Normality was assessed using the Shapiro–Wilk test, revealing non-normal distribution for most variables. Consequently, nonparametric methods were applied.

The Mann–Whitney U test was used to assess demographic and baseline scores between the VR-G and CG. Within-group pre–post changes (T0 vs. T1) were analyzed using the Wilcoxon signed-rank test for each group separately. In addition to statistical significance testing, effect sizes (Cohen’s d) were calculated for within-group pre–post changes to evaluate the magnitude of the observed effects. These calculations were performed separately for the VR group and the control group to provide a standardized measure of the impact of the interventions.

To investigate differences across age groups and gender, stratified analyses were performed by dividing the sample into subgroups based on age and gender, followed by within-subgroup comparisons using the Wilcoxon signed-rank test. This approach allowed for targeted examination of changes in emotional self-efficacy and cognitive outcomes within each age and gender subgroup.

Finally, Spearman’s correlation analysis was performed to evaluate the relationships between changes in emotional self-efficacy (positive and negative), cognitive function, and reductions in depressive (BDI) and anxiety (HRS-A) symptoms.

## 3. Results

**Figure 1:**
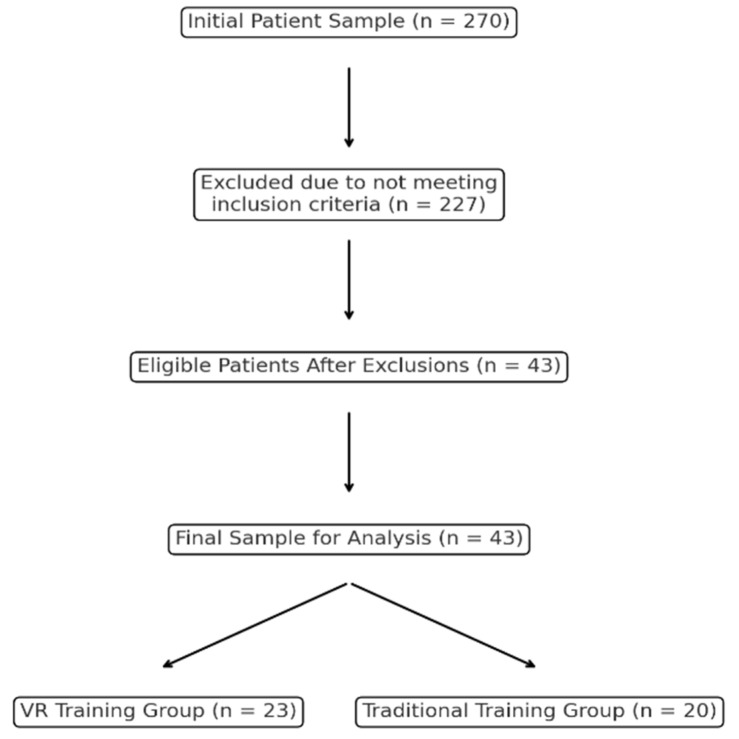
Flowchart of patient selection process.

The medical records of 270 patients with MS were included in the analysis using electronic recovery system data. After applying the inclusion and exclusion criteria, 227 patients were excluded due to factors such as age restrictions, recent relapses, or other criteria that did not let the patients use VR. This resulted in a final sample of 43 patients, divided into two groups: 23 patients in the VR-based training group and 20 in the traditional training group. The selection process is summarized in Figure 1.

The VR group consisted of 60.9% males and 39.1% females, with a mean age of 46.6 years (SD = 10.8), while the CG included 50% males and 50% females, with a mean age of 51.9 years (SD = 10.1). Education levels were balanced across groups, with the majority having over 8 years of education.

For more details, see Table 2.

Between-group comparisons at baseline (T0) confirmed that both groups were comparable across all measures, with no significant differences observed. Within-group comparisons of the VR group revealed significant improvements in several measures from T0 to T1 (Table 3). Specifically, negative emotional self-efficacy increased significantly (*p* = 0.011), indicating enhanced self-efficacy in managing negative emotions following the VR intervention. The depression (BDI) scores also showed a substantial decrease (*p* = 0.001), along with a reduction in anxiety (HRS-A) scores (*p* = 0.002), demonstrating the positive impact of VR on emotional health. Additionally, significant improvements were observed in RAO SRT-LTS (*p* < 0.001), RAO SRT-CLTR (*p* = 0.001), RAO SPART (*p* = 0.004), RAO PASAT 3 (*p* = 0.012), RAO PASAT 2 (*p* = 0.012), RAO SRT-D (*p* = 0.003), and RAO SPART-D (*p* = 0.025), reflecting enhanced cognitive function. In the control group, within-group analyses also indicated significant improvements in several outcomes. The depression (BDI) scores decreased significantly from T0 to T1 (*p* = 0.029), and anxiety (HRS-A) scores similarly declined (*p* < 0.001), suggesting that traditional therapy had positive effects on emotional health. Cognitive improvements were noted in RAO SRT-LTS (*p* = 0.002), RAO SRT-CLTR (*p* = 0.023), RAO SRT-D (*p* = 0.045), and RAO SPART-D (*p* = 0.019) (Table 3).

The effect sizes (Cohen’s d) for within-group pre–post changes were calculated to assess the magnitude of the observed improvements. In the VR group, large effect sizes were observed for measures such as depression (Cohen’s d = 1.45), negative emotional self-efficacy (Cohen’s d = 0.80), and verbal learning (Cohen’s d = 0.85), indicating the substantial impact of the VR-based intervention. In the CG, moderate to large effect sizes were found for anxiety (Cohen’s d = 0.89) and verbal memory (Cohen’s d = 0.72), demonstrating the effectiveness of traditional rehabilitation approaches for these domains. These findings provide a quantitative measure of the intervention’s impact, complementing the statistical significance results.

Spearman’s correlation analysis was conducted to assess the relationships between changes in emotional self-efficacy (both positive and negative), cognitive function, and reductions in depression (BDI) and anxiety symptoms (HRS-A).

**Figure 2:**
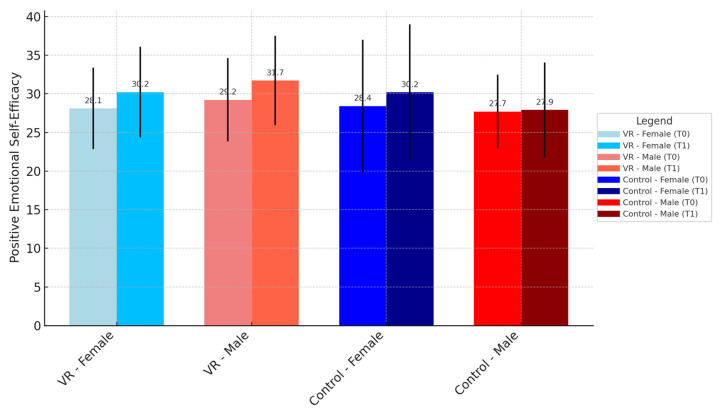
Gender differences in positive emotional self-efficacy at T0 and T1 in the virtual reality (VR) and control groups.

In the VR group, females showed a trend of improvement in positive emotional self-efficacy from T0 (median = 30.00, Q1 = 26.00, Q3 = 32.00) to T1 (median = 33.00, Q1 = 29.00, Q3 = 35.00) compared to males, who had a T0 median of 29.00 (Q1 = 25.00, Q3 = 31.00) and a T1 median of 31.00 (Q1 = 27.00, Q3 = 34.00). Although the trend indicates stronger improvement for females, this difference did not reach statistical significance (*p* = 0.124) (see Figure 2).

The figure illustrates gender-specific differences in positive emotional self-efficacy scores at baseline (T0) and post-intervention (T1) for both the VR and control groups. The data are presented separately for males and females within each group, highlighting trends in improvements over time. Bars represent median values, with error bars indicating the interquartile range (IQR). While both genders demonstrated improvements in positive emotional self-efficacy following the intervention, the VR-G, particularly females, exhibited a tendency for greater gains compared to the control group. These findings underline the potential influence of gender on the outcomes of VR-based rehabilitation.

**Figure 3:**
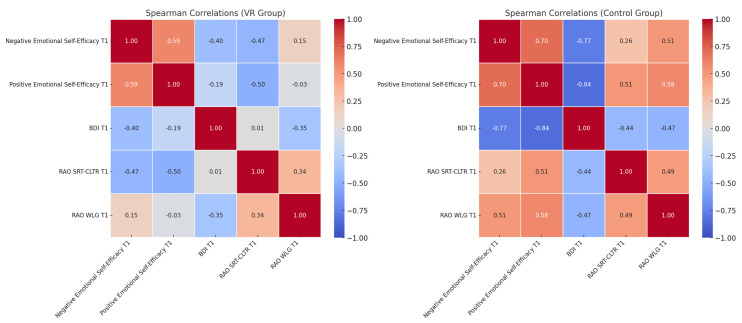
Spearman correlation heatmaps comparing the VR and control groups.

Moderate associations were observed between increased positive emotional self-efficacy and reductions in depressive symptoms (Spearman’s r = −0.33, *p* = 0.124) in the VR-G. This indicates a non-significant trend where gains in self-efficacy may relate to decreased depressive symptoms. Similar, though weaker, trends appeared in the CG. For cognitive outcomes, positive emotional self-efficacy improvements in the VR-G were significantly correlated with enhanced performance on long-term memory, specifically in the context of verbal learning (RAO SRT-CLTR: Spearman’s r = 0.31, *p* = 0.043). This suggests a link between emotional self-efficacy and verbal learning capabilities. Additionally, improvements in negative emotional self-efficacy were strongly associated with better working memory performance and verbal recall, as indicated by the RAO WLG score (Spearman’s r = 0.58, *p* < 0.001). In contrast, the CG showed no significant correlations between emotional self-efficacy and cognitive scores, highlighting a potential advantage of VR training in supporting cognitive improvement. For more details, see Figure 3.

The heatmaps illustrate the Spearman correlation coefficients between emotional self-efficacy (positive and negative), depressive symptoms (BDI T1), verbal learning (RAO SRT-CLTR T1), and working memory (RAO WLG T1) post-intervention. The left panel represents the VR group, showing stronger and more significant associations, particularly between emotional self-efficacy and cognitive measures. The right panel represents the control group, displaying weaker and non-significant correlations. Colors indicate the direction and strength of the correlation: red shades represent negative correlations; blue shades represent positive correlations; and the intensity of the color reflects the magnitude of the correlation (e.g., darker colors indicate stronger associations). Diagonal values are always 1.0, representing perfect correlations of variables with themselves.

## 4. Discussion

The findings from this study demonstrate that VR training can enhance emotional capacities by fostering positive emotional experiences and alleviating depressive symptoms. In the VR-G, significant improvements were observed in negative emotional self-efficacy (*p* = 0.011), depression (*p* < 0.001), and anxiety (*p* = 0.002), as well as in cognitive gains in verbal learning (*p* < 0.001) and working memory (*p* < 0.001). These positive outcomes were complemented by large effect sizes, indicating substantial clinical relevance. In contrast, the CG showed moderate improvements, particularly in emotional health and verbal memory. Furthermore, VR training appears to offer a slight advantage over traditional methods in promoting positive emotional self-efficacy, reducing depressive symptoms and improving cognitive outcomes. Indeed, this study highlights VR’s potential to empower patients in managing their emotional responses and self-efficacy. This aligns with recent findings on VR’s role in building emotional resilience by immersing patients in controlled and impactful environments. These latter enable the patients to better manage emotions, leading to improvements in mood and depressive symptoms [21,22]. This confidence in navigating emotional experiences in VR may also translate to daily life, helping patients maintain emotional well-being more effectively [23].

Moreover, our findings support the idea that the immersive and engaging VR environment facilitates self-reflection and controlled exposure to challenging scenarios [21,24]. The VR-G trend toward reduced depressive symptoms underscores VR’s unique potential as a powerful adjunct to traditional treatment, especially for building emotional resilience [6,25]. Such emotional resilience may be built through repetitive, realistic scenarios that safely simulate real-life challenges, helping users foster greater confidence in their emotional regulation abilities [22,23].

The immersive quality of VR differentiates it from traditional methods by better engaging users’ senses and attention. VR might potentially activate neural pathways related to emotional and cognitive processing. Indeed, neurophysiological research suggests that VR can stimulate areas like the prefrontal cortex, which are important for emotion regulation, decision-making, and resilience against depressive symptoms [21]. Further research is needed to better understand how these neural circuits are activated during VR sessions and their role in building ‘emotional scripts’ that could enhance confidence in managing real-life emotional situations [26].

Furthermore, VR facilitates exposure to therapeutic scenarios, known as “virtual exposure therapy”, which benefits patients by reducing anxiety and depressive symptoms without the pressures of the real-world contexts [24]. For patients with depression, this exposure can alleviate self-doubt and foster mastery, often reducing depressive symptoms [27,28]. Repeated VR emotional management exercises can lead to internalizing adaptive responses and building confidence in handling emotional challenges [23]. Parsons et al. [21] emphasized VR’s advantage in allowing patients to “rehearse” emotional responses, leading to significant gains in emotional resilience and reductions in depression severity. These findings support the view of VR as a flexible, interactive tool that complements traditional therapy by offering unique benefits in emotion management and self-efficacy.

### 4.1. Gender Differences in Emotional Self-Efficacy

Our data indicate that females in the VR-G may exhibit greater improvement in positive emotional self-efficacy scores compared to males, although the difference was not statistically significant. The observation agrees with existing research suggesting that females often engage more actively with emotional tasks in virtual environments. This could be due to differences in emotional processing, hormonal influences, and social learning [29].

Hormonal factors, such as the influence of estrogen and oxytocin, play a significant role in emotional sensitivity and social bonding, both of which may enhance engagement with emotional content in VR [30,31]. These hormones are linked to heightened activity in brain areas involved in emotional processing, such as the amygdala and prefrontal cortex [32]. Then, this hormonal predisposition may promote female positive self-reflection and responsiveness in supportive VR environments.

Neurological differences further contribute to gender-specific responses in VR interventions. Females tend to exhibit stronger interhemispheric connectivity in brain regions associated with emotional processing, empathy, and self-regulation [33]. This enhanced connectivity likely facilitates more effective emotional processing and regulation during reflective and interactive tasks, such as those provided in VR settings.

Additionally, the immersive nature of VR environments has been shown to enhance emotional engagement and responsiveness, particularly in scenarios designed to elicit empathy and reflection. Females, due to their heightened sensitivity to social and emotional cues, may benefit more from these immersive experiences [34].

These findings underscore the potential value of designing gender-sensitive VR interventions tailored to the distinct emotional processing styles of males and females. For females, incorporating scenarios that encourage interpersonal engagement and empathy may leverage their sensitivity to social cues and predisposition toward self-reflection [30,33,34]. Conversely, males may benefit from goal-oriented scenarios emphasizing problem-solving and assertive communication, aligning with their inclination toward outcome-focused tasks [31,35]. By accounting for these differences, VR interventions could more effectively optimize emotional and cognitive outcomes across genders.

### 4.2. Potential Neurophysiological Correlates and Cognitive Improvements in VR

VR training demonstrated significant correlations with emotional self-efficacy and cognitive outcomes. Improvements in positive emotional self-efficacy were correlated with enhanced verbal learning scores (RAO SRT-CLTR), while improvements in negative emotional self-efficacy were linked to better working memory (RAO WLG scores). These findings suggest that emotional regulation training in VR can positively impact cognitive domains, such as verbal learning and memory.

From a neurophysiological standpoint, VR may engage neural pathways involved in both emotional and cognitive functions, potentially activating areas such as the prefrontal cortex and hippocampus [36,37,38,39]. In addition, VR interventions have been shown to modulate activity in the amygdala and anterior cingulate cortex, regions implicated in emotional processing and cognitive control [38]. Evidence from functional MRI studies suggests that VR training can enhance neuroplasticity in these regions by promoting the integration of sensory, emotional, and cognitive processes [38,39]. However, further research is needed to clarify how these brain regions are specifically activated during VR sessions and their role in cognitive and emotional outcomes.

These correlations suggest that VR training might offer cognitive benefits that are not typically observed with traditional rehabilitation methods. For instance, the interactive and immersive nature of VR engages attentional and executive processes in a dynamic manner [38], which could facilitate better retention and transfer of emotional regulation skills to real-life contexts. The relationship between emotional self-efficacy and cognitive gains may reflect an integrated neurophysiological mechanism in which VR training helps build emotional resilience, which in turn may support cognitive function [36,37,38,39].

### 4.3. Comparison Between VR and Traditional Rehabilitation Methods

Traditional rehabilitation methods, including physical therapy and cognitive training, have proven effective in addressing motor and cognitive impairments in MS patients [8]. However, they often rely on repetitive, passive exercises, which may limit patient engagement. In contrast, VR rehabilitation represents a significant advancement, offering a more interactive and immersive approach [10,20,23]. By combining physical, cognitive, and emotional interventions, VR allows for more personalized and engaging treatment plans. Unlike conventional rehabilitation, VR interventions provide real-time feedback, encourage active participation, and can be customized to individual progress [20]. Our study suggests that VR has the potential to enhance both emotional self-efficacy and cognitive function, presenting a novel alternative to traditional therapies. This approach is particularly valuable in addressing emotional and cognitive challenges—areas often underrepresented in conventional rehabilitation strategies.

### 4.4. Implications and Future Directions

Building on the benefits highlighted in the previous section, VR training may offer a unique advantage by creating realistic yet controlled environments for patients to practice and refine emotional responses. VR facilitates improvements in self-efficacy and emotional regulation, enabling patients to confront emotional challenges safely and foster confidence that can generalize to real-life situations [23]. Furthermore, interactive feedback in VR accelerates learning and emotional resilience by enabling patients to observe and adjust their responses in real-time [24]. The correlations observed between emotional self-efficacy and cognitive function suggest that VR has the potential for multidimensional benefits. Future research should investigate the long-term effects of VR on self-efficacy and explore neurophysiological changes through EEG or fMRI to better understand the underlying neural mechanisms [24,36]. Additionally, examining gender-specific adaptations will help refine VR protocols, ensuring maximum benefit for diverse patient populations.

Another aspect that warrants further exploration is how different VR environments, such as naturalistic versus gamified settings, might influence emotional and cognitive rehabilitation outcomes. Naturalistic environments, designed to mimic real-world settings, could facilitate relaxation and emotional regulation by offering a sense of familiarity and immersion. On the other hand, gamified settings, with their interactive and reward-driven features, are likely to enhance engagement, motivation, and adherence, particularly in tasks targeting attention, executive functions, and problem-solving [40,41]. While the current study utilized a semi-immersive 2D environment tailored to individual patient needs, future research could investigate whether integrating different approaches might optimize therapeutic outcomes. Such investigations could help determine how varying levels of immersion and interactivity contribute to specific rehabilitation goals and patient preferences, ultimately guiding the development of more personalized and effective VR interventions.

Finally, future research could explore the integration of VR interventions with pharmacological treatments to investigate potential combined therapeutic strategies. While VR effectively targets cognitive, emotional, and motivational aspects, pharmacological treatments address neurochemical imbalances, offering complementary benefits [42]. For instance, in neurological conditions such as multiple sclerosis, disease-modifying therapies can slow disease progression and manage inflammatory processes, while VR addresses cognitive and physical rehabilitation needs [43,44,45,46]. Such multimodal approaches could maximize therapeutic outcomes by leveraging the strengths of both methods. Studies on the timing, dosage, and mechanisms of this integration would provide valuable insights for personalized treatments.

### 4.5. Novelty, Strengths, and Limitations

This study provides several novel insights into the use of VR in the rehabilitation of patients with MS. Unlike previous research, which primarily focused on motor rehabilitation, this study explores the integration of emotional self-efficacy and cognitive outcomes, offering a more holistic perspective on the therapeutic potential of VR. Additionally, the analysis highlights trends in gender-specific responses, particularly a tendency for females to exhibit greater improvements in positive emotional self-efficacy. This represents a valuable direction for future research, especially in tailoring gender-sensitive rehabilitation interventions.

Another innovative aspect of this study is the identification of correlations between emotional and cognitive improvements, suggesting the interconnected nature of these domains and the potential for VR to address them simultaneously.

Despite these strengths, the study has several limitations that should be acknowledged. First, the relatively small sample size may limit the generalizability of the findings, as it reduces statistical power, particularly in detecting subtle effects across different demographic groups such as age and gender. Additionally, the non-randomized design means that, although no significant differences were found between the VR and control groups, the potential for selection bias remains, as pre-existing differences between the groups could influence the results, despite efforts to control for baseline characteristics. Although nonparametric methods were appropriate for the data distribution, they may limit the comparability of these findings with other studies employing parametric analyses.

Another limitation is the self-report nature of emotional self-efficacy measures, which are subject to social desirability bias and may not fully capture the complexity of emotion regulation abilities in real-life contexts. Additionally, while we examined correlations between cognitive improvements and emotional self-efficacy, the cross-sectional nature of these analyses precludes any causal inferences regarding the impact of VR on other functional domains. Finally, the study did not incorporate advanced neurophysiological measures such as EEG or fMRI to directly assess changes in brain activity, which could provide more precise information about the neural mechanisms underlying the effects of VR.

Future studies with larger randomized samples, extended follow-up periods, and neuroimaging assessments would strengthen the validity of these findings and provide a more comprehensive understanding of the long-term therapeutic potential of VR in MS rehabilitation.

## 5. Conclusions

VR offers a promising alternative and complementary tool for rehabilitation, enhancing emotional self-efficacy and adaptive emotion management. By bridging skill acquisition in clinical settings with real-life applications, VR creates a dynamic and flexible approach to empower patients in managing emotional and cognitive challenges.

## Figures and Tables

**Table 1:** Comparison of rehabilitation protocols for experimental (VRRS) and control groups.

Training Component	Virtual Reality Group	Control Group (CG)
Cognitive Rehabilitation	VRRS (Virtual Reality Rehabilitation System)	Traditional paper-and-pencil tasks
Session Frequency	3 times per week	3 times per week
Session Duration	60 min	60 min
Therapy Focus	2D and 3D VR-based exercises for cognitive domains (memory, attention, planning)	Standard exercises for memory, attention, planning

This table outlines the key components of the rehabilitation protocols for the experimental group using the Virtual Reality Rehabilitation System (VRRS) and the control group (CG) following traditional methods. Details include the cognitive rehabilitation approach, session frequency, duration, and therapy focus.

**Table 2:** Demographic and clinical characteristics of patients.

Demographic Variables	VR Group (*n* = 23)	Control Group (*n* = 20)	*p*-Value
Gender Male Female	14 (60.9%) 9 (39.1%)	10 (50.0%) 10 (50.0%)	0.683
Age (years)	46.6 ± 10.8	51.9 ± 10.1	0.104
Education Level			
Elementary School	2 (8.7%)	3 (15.0%)	0.868
Inferior School	3 (13.0%)	4 (20.0%)	0.840
High School	10 (43.5%)	7 (35.0%)	0.799
University	8 (34.8%)	6 (30.0%)	0.994
Disease Duration (years)	8.3 ± 4.5	8.0 ± 4.7	0.99
EDSS (mean ± SD)	4.5 ± 1.1	4.5 ± 1.1	1.00

This table summarizes the demographic and clinical characteristics of the study participants. Variables include age, gender distribution, education level, and baseline clinical measures (e.g., BDI, HRS-A). Continuous variables are reported as mean ± standard deviation (SD) or median (interquartile range, Q1–Q3) based on data distribution, while categorical variables are presented as frequencies and percentages. Legend: Expanded Disability Status Scale (EDSS).

**Table 3:** Wilcoxon rank test for pre–post changes (T0–T1): median, interquartile range (Q1–Q3), and *p*-values.

Outcome Measure	VR Group		*p*-Value (T0 vs. T1)	Control Group		*p*-Value (T0 vs. T1)
	T0 (Median [Q1, Q3])	T1 (Median [Q1, Q3])		T0 (Median [Q1, Q3])	T1 (Median [Q1, Q3])	
Positive Emotional Self-Efficacy	30.00 [25.50, 32.50]	33.00 [29.00, 35.00]	0.076	29.00 [23.50, 33.25]	29.50 [23.75, 35.00]	0.199
Negative Emotional Self-Efficacy	26.00 [23.00, 29.50]	31.00 [24.00, 35.00]	<0.01 *	28.00 [21.00, 32.00]	28.50 [23.75, 34.00]	0.162
Depression (BDI)	17.00 [10.00, 21.00]	9.00 [3.50, 11.50]	<0.001 **	12.50 [7.75, 21.25]	8.00 [3.75, 17.25]	0.029 *
Anxiety (HRS-A)	13.00 [9.00, 22.50]	6.00 [2.50, 20.00]	0.002 *	18.50 [12.00, 24.00]	12.00 [3.75, 16.25]	<0.001 **
RAO SRT-LTS	28.37 [19.16, 35.16]	40.16 [31.28, 49.66]	<0.001 **	24.36 [15.76, 30.92]	32.60 [26.91, 37.67]	0.002 *
RAO SRT-CLTR	21.99 [13.66, 28.97]	27.08 [21.84, 35.91]	<0.001 **	15.49 [10.62, 24.21]	24.34 [18.80, 27.85]	0.023 *
RAO SPART	14.62 [10.91, 20.05]	20.62 [14.28, 23.04]	0.004 *	15.78 [11.53, 17.12]	17.67 [12.16, 23.31]	0.097
RAO SDMT	31.53 [20.45, 38.83]	31.53 [24.94, 39.88]	0.080	32.45 [19.49, 39.74]	36.89 [18.42, 46.38]	0.121
RAO PASAT 3	26.59 [18.58, 35.83]	28.90 [20.57, 41.94]	0.012 *	34.97 [20.33, 49.27]	33.79 [23.23, 47.98]	0.737
RAO PASAT 2	21.87 [15.29, 26.91]	21.50 [17.98, 31.40]	0.012 *	22.89 [16.47, 35.33]	24.83 [19.22, 32.66]	0.638
RAO SRT-D	5.87 [3.88, 7.08]	7.88 [6.88, 8.88]	0.003*	4.88 [3.88, 6.18]	6.38 [4.63, 7.64]	0.045 *
RAO SPART-D	5.56 [4.25, 7.48]	6.92 [4.56, 8.74]	0.025 *	4.92 [3.56, 5.71]	5.48 [4.40, 7.92]	0.019 *
RAO WLG	20.88 [14.00, 24.50]	20.88 [17.50, 27.46]	0.115	22.12 [18.69, 25.31]	20.50 [15.31, 24.29]	0.124

This table presents the results of the Wilcoxon rank test, comparing pre- (T0) and post-intervention (T1) measures. For each variable, the median values and interquartile ranges (Q1–Q3) are reported alongside the corresponding *p*-values. Statistically significant changes (*p* < 0.05) are highlighted to indicate meaningful differences over time. Statistically significant differences (*p* < 0.05) are indicated with a single asterisk (*), while those with even greater significance (*p* < 0.001) are indicated with two asterisks (**). Legend: Beck Depression Inventory (BDI); Hamilton Rating Scale for Anxiety (HRS-A); Rao Brief Repeatable Battery of Neuropsychological Tests for Multiple Sclerosis: Rao Paced Auditory Serial Addition Test—2 s (RAO PASAT 2), Rao Paced Auditory Serial Addition Test—3 s (RAO PASAT 3), Rao Selective Reminding Test—Consistent Long-Term Retrieval (RAO SRT-CLTR), Rao Selective Reminding Test—Delayed (RAO SRT-D), Rao Selective Reminding Test—Long-Term Storage (RAO SRT-LTS), Rao Spatial Recall Test (RAO SPART), Rao Spatial Recall Test—Delayed (RAO SPART-D), Rao Symbol Digit Modalities Test (RAO SDMT), Rao Word List Generation (RAO WLG).

## Data Availability

The data supporting the findings of this study are available upon request from the corresponding author. The data are not publicly available due to privacy or ethical restrictions.
